# Cost Analysis of Orthoptist-Led Neurofibromatosis Type 1 Screening Clinics

**DOI:** 10.22599/bioj.288

**Published:** 2023-04-10

**Authors:** Navdeep Kaur, Catherine Lewis, Sandra Staffieri, Jonathan Ruddle, Ilias Goranitis, Jay Stiles, Gabriel Dabscheck

**Affiliations:** 1 Department of Ophthalmology, The Royal Children’s Hospital, Victoria, AU; 2 Centre for Eye Research Australia, Royal Victorian Eye and Ear Hospital, AU; 3 Murdoch Children’s Research Institute, Melbourne, AU; 4 Health Economics Unit, Centre for Health Policy, Melbourne School of Population and Global Health, The University of Melbourne, AU; 5 Department of Neurology, The Royal Children’s Hospital, Victoria, AU

**Keywords:** orthoptist-led, NF1, optic pathway glioma, cost-analysis, health economics, costing-study

## Abstract

**Purpose:**

To conduct a costing study comparing orthoptist-led with consultant-led clinics screening for optic pathway gliomas (OPGs) in children with neurofibromatosis Type 1 (NF1) attending the Royal Children’s Hospital (RCH), Melbourne.

**Methods:**

Patients with NF1 examined in the orthoptist-led NF1 screening clinic and/or consultant-led clinics during the study period were identified. The workflow management software Q-Flow 6® provided data documenting patient’s time spent with the orthoptist, nurse, and ophthalmologist. Time points were converted into minutes and multiplied by the cost-per-minute for each profession. A bottom-up micro-costing approach was used to estimate appointment level costs. Bootstrap simulations with 1000 replications were used to estimate 95% confidence intervals (CIs) for the difference in mean appointment time and cost between clinics.

**Results:**

Data for 130 consultant-led clinic appointments and 234 orthoptist-led clinic appointments were extracted for analysis. The mean time per appointment for the consultant-led clinic was 45.11 minutes, and the mean time per appointment for the orthoptist-led clinic was 25.85 minutes. The mean cost per appointment for the consultant-led clinic was A $84.15 (GBP £39.60) compared to the orthoptist-led clinic at A $20.40 (GBP £9.60). This represents a mean reduction of 19.25 minutes per appointment (95% CI, –24.85 to –13.66) and a mean reduction of A $63.75 (GBP £30.00) per appointment (95% CI, (A $-75.40 to $-52.10 [GBP £ -35.48 to £ -24.52]).

**Conclusion:**

An orthoptist-led clinic screening for OPGs in patients with NF1 can be a more cost-efficient model of care for ophthalmic screening in this patient group.

## Introduction

Neurofibromatosis Type 1 (NF1) is a neurogenetic disorder that affects approximately 1 in 3600 individuals worldwide (Cimino & Gutmann, 2018, Evans et al., 2010). Individuals with NF1 are prone to the development of pilocytic astrocytoma, the most common of which are optic pathway gliomas (OPG) (Campen & Gutmann 2018). An OPG is classified by the World Health Organization (WHO) as a grade 1 neoplasm that presumably arises from supporting astrocytes in the optic nerve (Kleihues et al. 2002). In patients with NF1, gliomas most frequently occur within the anterior optic pathway and brainstem (Guillamo et al. 2003).

OPGs occur in approximately 15% of patients with NF1 (Fisher et al. 2012), with the greatest risk of developing within the first six years of life (Listernick et al. 1994). The median age for an OPG diagnosis is 2.66 years (range 0.36–15.8), with approximately 40% of patients with NF1 being asymptomatic (Fisher et al. 2012). Symptoms of an OPG include decreased visual acuity, abnormal pupillary functions, decreased colour vision, optic atrophy, reduced visual field, and proptosis (Suharwardy & Elston 1997). The main objective in the management of patients with NF1 and an OPG is to prevent decreased visual acuity and neurological damage (Fried et al. 2013). Vision reduction can be unilateral or asymmetrical and children may not complain of vision problems as they readily adapt to this change. Therefore screening for an OPG in patients with NF1 is crucial (Thiagalingam et al. 2004).

In 1997, the National Institutes of Health OPG Task Force outlined the recommendations for screening, follow-up, and treatment of children with NF1 without an OPG. The ophthalmic assessment at a minimum required visual acuity, visual fields, colour vision, as well as fundus and slit-lamp examination. Pupillary reflex, cycloplegic refraction, and ocular motility assessment were also recommended (Listernick et al. 1997).

In 2015, Caen et al. evaluated the use of these guidelines and reported variations in OPG screening parameters across multiple centres internationally for patients with NF1. The study highlighted the lack of uniformity in ophthalmic screening for an OPG in patients with NF1 regarding frequency and duration of ophthalmic reviews, as well as variations in ocular assessments (Caen et al. 2015). The majority of centres surveyed did not perform colour vision, visual fields, or slit-lamp examination as recommended by the National Institutes of Health OPG Task Force (Listernick et al. 1997).

Following this review, recommendations made by Caen et al. (2015) suggested six-monthly ophthalmic screening until the age of six years, then yearly examinations from six years until adulthood. At a minimum the clinical assessment should include: visual acuity assessment, pupillary responses, and fundoscopy. Neuroimaging, whilst a useful modality to diagnose an OPG, is only indicated in the presence of neurological or ophthalmic abnormalities (King et al. 2003).

Ophthalmic screening for OPGs is traditionally conducted in the context of a comprehensive ophthalmic examination. To screen children with NF1 for ocular signs of an OPG, we aimed to compare the cost of a novel orthoptist-led NF1 clinic in our centre to a consultant-led clinic. Through conducting a time and motion study which involves direct and continuous analysis of time through a process/event (Lopetegui et al. 2014), we assessed the flow of patients undergoing NF1 ophthalmic screening through each model, and compared the mean appointment cost per patient.

## Methods

**Figure 1:**
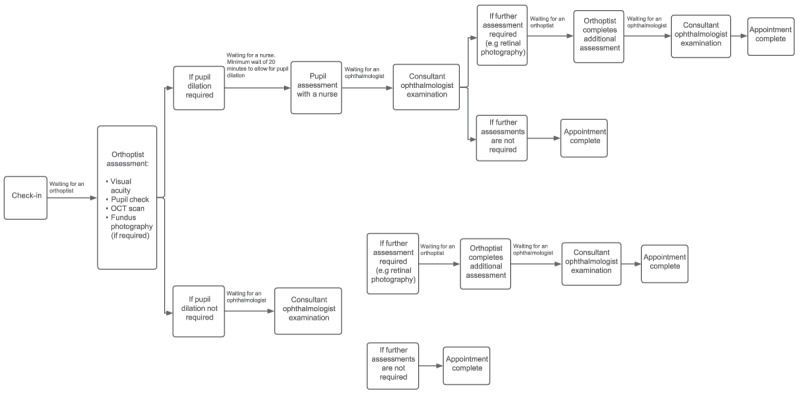
Patient flow through consultant-led clinic.

Historically, at the RCH, Melbourne, Victoria, all patients with NF1 requiring screening for OPGs were examined in consultant-led ophthalmology outpatient clinics. Our consultant-led clinic comprises of a service in which the ophthalmologist has overall responsibility for the service, actively conducts a clinical examination and provides treatment. This comprehensive assessment included examination by an orthoptist (assessment of parameters of visual function including visual acuity, visual fields, and colour vision assessment) and instillation of dilating eye drops. Pupillary assessment by a nurse followed and instillation of the second dose of eye drops, if mydriasis was incomplete. Lastly, a fundus examination and cycloplegic refraction were completed by the consultant ophthalmologist. If the consultant required additional assessments, patients were re-routed to the orthoptist. Once completed, the patients waited to see the ophthalmologist again to complete their appointment (Figure 1).

**Figure 2:**
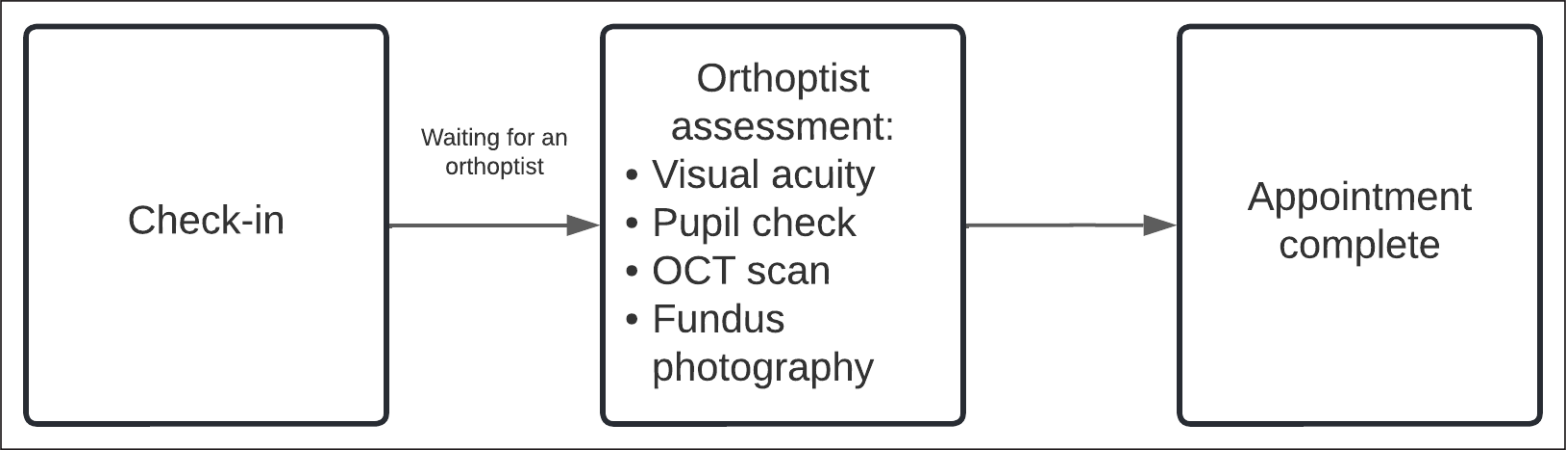
Patient flow through an orthoptist-led clinic.

Supported by evidence-based research (Caen et al. 2015), and through the collaborative efforts between the departments of ophthalmology and neurology at our institution, an assessment protocol was developed implementing an orthoptist-led screening clinic for children under 18 years of age, diagnosed with NF1 with no known OPGs. Our orthoptist-led clinic is a service in which the orthoptist has overall responsibility for the service delivered and conducts all the clinical assessments related to the appointment. Implemented in March 2016, the orthoptist-led NF1 clinic protocol (Supplementary Materials A) retained all assessments performed in the consultant-led clinic, followed current OPG screening recommendations (Caen et al. 2015), and did not require: a dilated eye examination, a nurse or a consultant ophthalmologist, and included an Optical Coherence Tomography (OCT) Retinal Nerve Fibre Layer (RNFL) scan (Figure 2). Although the National Institutes of Health OPG task force have recommended assessments such as colour vision and visual field testing for the detection of an OPG in patients with NF1, these were not included in our orthoptist-led NF1 clinic (Listernick et al. 1997). Whilst visual field and colour vision are also potential markers of functional decline, they typically occur concurrently with visual acuity loss. In a large retrospective study, initiation of therapy for an OPG was mostly indicated by a decrease in vision. Therefore, visual acuity was deemed the most reliable functional measure of vision for patients with NF1 with an OPG (Fisher et al. 2013).

The orthoptist-led NF1 clinic appointments are scheduled on the same day as the patient’s neurology appointment, reducing the frequency of hospital visits. At the conclusion of each patient’s assessment, the results are tabulated and made available electronically for review by the ophthalmology and neurology consultant staff.

All new diagnoses of OPGs referred to oncology were reviewed on a weekly basis during an oncology multidisciplinary meeting. This was to confirm that no patients referred to oncology via parallel health services had been examined in the NF1 screening clinic and their diagnosis of an OPG had been missed.

### Study Cohort and Data Collection

All patients examined in the orthoptist-led NF1 clinic between 4^th^ May 2016 to 31^st^ October 2018 were identified using the institution’s electronic medical records (EMR) search function. Patient appointments for those seen in the consultant-led clinic prior to the orthoptist-led NF1 clinic between 27^th^ June 2014 and 20^th^ March 2018 were identified as the comparator. An overlap of study cohorts resulted when patients with NF1 with no known OPG transitioned from the consultant-led clinic into the newly established orthoptist-led clinic. Some patients were reviewed in the consultant-led clinic and referred to the orthoptist-led screening clinic after a normal routine assessment.

Patients with NF1 who attended a consultant-led clinic appointment that was unrelated to their NF1 diagnosis (e.g., strabismus assessment) were excluded from this study.

### Data Extraction

Workflow management software Q-Flow 6®, Q-nomy Inc. FL, USA, 2020, is utilised at our institution. This software records each patient’s check-in time, duration of consultation with each clinician (orthoptist, nurse, ophthalmologist) as well as waiting and departure times. On presentation to reception, the reception staff check the patient in on Q-Flow 6®, which is their first time-stamp point. The orthoptist then uses the Q-Flow 6® software to send a text message to the parent calling the patient to their room, creating the second time-stamp point. This process continues with each health professional encountered until the appointment is completed in both the orthoptist-led and consultant-led models of care. The time between each encounter with a professional is recorded in Q-flow 6® in minutes and seconds. For each patient appointment in the study cohort, data time-points relating to each visit and clinician encounter were extracted from Q-Flow 6® by the Australian licensor NEXA, NEXA Group 2006, for analysis. The time spent in minutes with each clinician for both models of care was rounded to the nearest minute and a mean time was derived.

### Microcosting Approach

A bottom-up microcosting approach was adopted to estimate direct health care costs. Appointment level costs were according to clinician and clinic type. Clinician salary cost per minute was derived from the respective professional enterprise agreements (Fair Work Commission Australia 2018; Fair Work Commission Australia 2016; Fair Work Commission Australia 2017). Salary cost per minute was then multiplied by the time spent with each clinician. Total costs per appointment were then summed and a mean appointment cost per clinic was calculated.

### Statistical Analysis

Bootstrap simulations with 1000 replications were used to estimate 95% confidence intervals (CIs) for the difference in mean appointment time and cost between clinics. A mean purchasing price parity from 2014–2018 was derived for a currency conversion with subsequent costs listed in Australian dollars and Pounds Sterling (Purchasing Power Parities). All statistical analyses were performed in Stata Version 16.1 (StataCorp, College Station, TX, USA).

This study adhered to the tenets of the Declaration of Helsinki and was approved by the RCH Human Research Ethics Committee (HREC- 2019.032).

## Results

During the study periods for each clinical model, 234 appointment encounters for 130 patients attending the orthoptist-led clinic and 130 appointments for 80 patients attending the consultant-led clinic were identified and analysed.

As shown in Table 1, the mean time with the orthoptist in the orthoptist-led model of care is longer than that in the consultant-led model (25.85 ± 28.2; 19.99 ± 14.15 respectively). However, the total mean time per appointment is lower in the orthoptist-led clinic compared to the consultant-led clinic.

**Table 1:** Mean time in minutes spent with each clinician for consultant-led and orthoptist-led models of care encounters.


CLINICIAN	CONSULTANT-LED (N = 130)	ORTHOPTIST-LED (N = 234)	DIFFERENCE (CONSULTANT LED – ORTHOPTIST LED)		

	MEAN (SD) MINUTES	MEAN (SD) MINUTES	MEAN TIME DIFFERENCE	NORMAL-BASED 95% CIS	NORMAL-BASED 95% CIS

Consultant	22.31 (21.93)	N/A			

Nurse time	2.81 (2.49)	N/A			

Orthoptist time	19.99 (14.15)	25.85 (28.2)			

**Mean time per appointment**	**45.11 (26.04)**	**25.85 (28.2)**	**19.25**	**24.85**	**13.66**


*Abbreviations*: SD = Standard deviation; N/A = Not applicable; CI = Confidence Interval.

**Figure 3:**
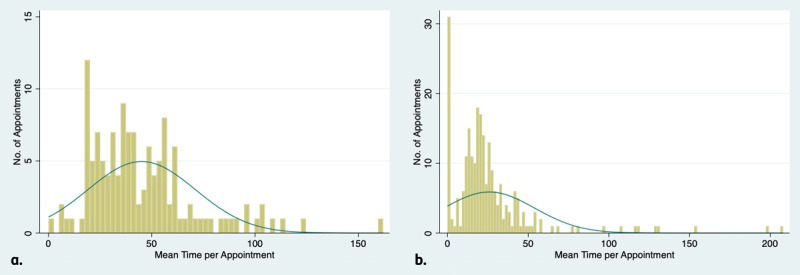
**(a)** Distribution of time consultant-led clinic. **(b)** Distribution of time orthoptist-led clinic.

For each clinic, the distribution of time per appointment demonstrated a heavy right skew for both consultant-led (Figure 3a), and orthoptist-led clinics (Figure 3b), although orthoptist-led clinics overall required less total time. The orthoptist-led clinic had a longer tail. However, for both clinics, some appointments experienced values several times the mean.

As demonstrated in Table 2, the mean cost per appointment for the consultant-led clinic was A $84.15 (GBP £39.60) and the mean cost per appointment for the orthoptist-led clinic was A $20.40 (GBP £9.60). We examined the difference in mean cost per appointment between clinics. Compared to the consultant-led clinic, the orthoptist-led clinic resulted in a reduction of A $63.75 (GBP £30.00) per appointment (95% CI (A $-75.40 to $-52.10 [GBP £ -35.48 to £ -24.52]).

**Table 2:** Mean cost in Australian dollars (A$) and pound sterling (GBP£) per appointment by health professional.


	CONSULTANT-LED (N = 130)		ORTHOPTIST-LED (N = 234)		DIFFERENCE (CONSULTANT LED – ORTHOPTIST LED)				
		
	MEAN (SD) A$	MEAN (SD) GBP£	MEAN (SD) A$	MEAN (SD) GBP£	MEAN COST DIFFERENCE	NORMAL-BASED 95% CIS	NORMAL-BASED 95% CIS	SALARY/PER MINUTE A$ (GBP£)	SOURCE(A$)

Consultant cost	66.49 (65.35)	31.29 (30.75)	N/A	N/A				2.98 (1.40)	AMA Victorian Public Health Sector- Medical Specialists Enterprise Agreement 2018–2021 (Fair Work Commission Australia 2018)

Nurse cost	1.88 (1.67)	0.88 (0.79)	N/A	N/A				0.67 (0.32)	Nurse and Midwives (Victorian Public Health Sector) (Single Interest Employers) Enterprise Agreement 2016–2020 (Fair Work Commission Australia 2016)

Orthoptist cost	15.77 (11.16)	7.42 (5.25)	20.40 (22.25)	9.60 (10.47)				0.79 (0.37)	Allied Health Professionals (Victorian Public Health Sector) Single Interest Enterprise Agreement 2016–2020 (Fair Work Commission Australia 2017)

**Mean cost per appointment (SD)**	**84.15 (66.18)**	**39.60 (31.14)**	**20.40 (22.25)**	**9.60 (10.47)**					**NA**

**Cost difference (95% CI) A$**						**–63.75**	**–75.40**	**–52.10**	

**Cost difference (95% CI) GBP£$**						**–30.00**	**–35.48**	**–24.52**	


*Abbreviations*: n =number of observations; NA = not applicable; SD = standard deviation.

**Figure 4:**
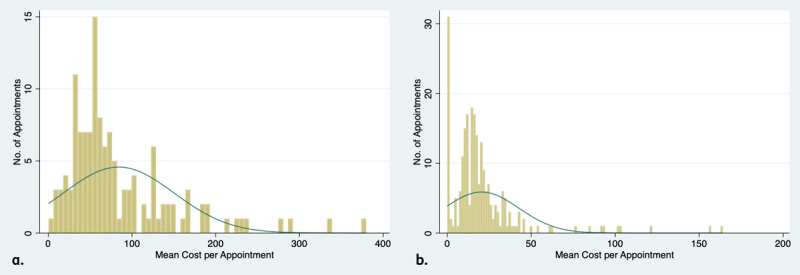
**(a)** Distribution of cost consultant-led clinic. **(b)** Distribution of cost orthoptist-led clinic.

Much like the distribution of time per appointment in 3a and 3b, the distribution of costs per appointment followed similar heavily right-skewed distributions (Figures 4a and 4b). Unlike the distribution of appointment times, the consultant-led clinic demonstrated a longer tail. Both clinics included some appointments with costs several times the mean.

Overall, the analyses in differences in time and costs between the models of care demonstrate that the findings can be extrapolated to larger settings.

There were no patients who attended the orthoptist-led screening clinic that were diagnosed with an OPG during the study period or presented to oncology with an OPG that was missed in the orthoptist-led NF1 screening clinic.

## Discussion

Our study findings describe the cost benefit of an orthoptist-led NF1 screening clinic. When compared to the consultant-led clinic, the orthoptist-led clinic benefits included both a 20-minute shorter total examination time (excluding wait time), and nearly A$64 (GBP £30) reduction in cost per patient. The significant difference in cost between both models of care results from the orthoptist-led clinic not requiring an ophthalmologist or nurse. The consultant cost represented the majority of the mean patient cost in the consultant-led clinic and was the key driver in the difference between the costs per clinic. These results demonstrate a lower cost alternative care model providing an opportunity for judicious use of health system resources (Gammie et al. 2016).

The orthoptist appointment time on average was 5.86 minutes longer in the orthoptist-led clinic compared to the consultant-led clinic. As the orthoptist is the sole clinician in the orthoptist-led clinic, they are required to complete the same clinical assessments performed in the consultant-led clinic appointments, as well as summarise the appointment findings to the patients and parents.

From a patient’s perspective, the opportunity cost of their time is also significant. Spending 20 minutes less at the clinic with a clinician is likely to be welcomed by patients and their families (Ballantyne et al. 2019). Shorter appointment times without compromising the quality of care encourages families to accept future allied health-led clinic appointments (Pokorny et al. 2021). As the orthoptist-led NF1 clinic appointments are scheduled on the same day as the patient’s neurology appointment, costs, and time associated with hospital appointments for the patient and family are reduced. From a hospital’s perspective, our findings support the allocation of resources to an allied health-led clinic as a judicious use of hospital resources. Therefore, allied health-led clinics are indicative of cost and time-efficient alternate care models from both a healthcare and patient viewpoint.

Allied health-led clinics can improve patient outcomes, allow for timely care, and provide positive patient experiences, alongside greater compliance with national guidelines (Bouraoui et al. 2020; Philip 2015). An optometrist-led model of care in the assessment and monitoring of naevomelanocytic lesions has shown to be a successful alternative care model compared to a traditional consultant-led model (Karthikeyan et al. 2021). Audit results from this study reported high patient satisfaction; all patients were happy with their clinical experience with 95% of patients happy to continue care with an allied health professional. Mutual agreement of patient diagnosis and follow-up between the allied health and medical professionals in the alternate care model was seen for the majority of patients. Their findings suggest shared knowledge and clinical judgement between the professions, and highlights the safety of alternative care models with high patient satisfaction, further supporting the autonomous work of allied health professionals.

Nurse-led follow-up clinics have also been accepted across numerous clinical settings in ophthalmology including the surveillance of ocular tumours which require long-term monitoring (Sandinha et al. 2012), diabetic screening (Kirkwood et al. 2006), and screening for retinopathy of prematurity (Tram et al. 2021). Tram et al. (2021) described a nurse-led retinopathy of prematurity clinic in which fundus photos are taken by the nurse and assessed offline by an ophthalmologist. An assessment of the fundus offline reduced an ophthalmologist’s time spent screening a patient without reducing the number of patients being screened (Tram et al. 2021). At a time when demands on ophthalmic services are increasing, our study findings are encouraging and promote areas where the scope of practice for orthoptists, allied health, and nursing can be expanded.

Although multiple studies have assessed the clinical effectiveness of an alternative model of care, they lack a costing component where monetary difference is assessed (Kirkwood et al. 2006). NF1 is a condition with well-published surveillance guidelines, optimising early diagnosis and management of complications (Caen et al. 2015; Listernick et al. 1997). Although screening for an OPG in children with NF1 is accepted best practice, the current model of screening for OPGs in children with NF1 is time-consuming and costly (Blazo et al. 2004). Health economic evaluations provide information for analysing the cost and benefit of healthcare interventions in the presence of limited resources and increased demand (Atik et al. 2020). Therefore, completing a costing study on an allied health clinic provides a foundation for future clinicians to advocate for alternate models of care within their centres, clinics, and hospitals. Presenting a more cost-efficient model of care can promote autonomy within the orthoptics profession and can assist in the evolution of the profession. Results from this study can be transferrable across multiple health professions within the private and public health sectors worldwide. In health systems where orthoptists or nursing staff are not available, there is potential to upskill a layperson who can follow the NF1 screening protocol as administration of dilating drops is not required.

Our findings demonstrate a gain in technical efficiency and reveal an opportunity to screen a greater number of patients. Consultant-led clinic appointments previously allocated to screening for an OPG in patients with NF1 can now be utilised by other patients, reducing delays between appointments (Stute et al. 2021). The benefits of surveillance and screening clinics in facilitating earlier detection of pathology while simultaneously reducing the costs associated is well understood (Watts et al. 2015).

The strength of our study was the robust time-and-motion analysis generated using the program Q-Flow 6® along with the bottom-up microcosting methodology we applied to resource utilisation. Combined, these techniques result in an accurate representation of the clinics from a health care perspective (Xu et al. 2014). Although our study had positive and valuable findings, there were multiple limitations which included the inability to adopt a societal perspective. Future research should consider capturing indirect costs related to patient travel time and their opportunity costs. Secondly, the underlying data was heavily skewed and with unequal variance. We attempted to address this with bootstrapping. Bootstrapping is a useful non-parametric technique that uses repeated and random samples of the same size as the original sample. These are drawn with replacement from the data to estimate the shape of a statistic’s sampling distribution empirically (Campbell & Torgerson 1999). Lastly, the patient’s time spent in the consultant-led clinic was likely an underestimation. As these patients usually require eye drops, this involves them waiting for a minimum of 20 minutes for the cycloplegic effect. For some patients, this period is even longer. The nurses are responsible for assessing pupil dilation and deciding when the pupil is sufficiently dilated to be routed to the consultant queue. The time between the orthoptist and the consultant where the patient is under the care of a nurse was captured, however, modified for the analysis in a way that it was not truly representative. As this was a costing study, we only included the time the patient was physically with the nurse and estimated this to be five minutes. Future research could consider a societal perspective and fully capture the total patient wait time. Regardless, our results demonstrate an improvement in efficiency as compared to the consultant-led clinic.

For any care pathway, patient safety and clinical outcomes remain a priority. Future research could consider capturing patient outcomes, wait-time between clinicians and patients who failed the orthoptist-led clinic and required ophthalmology reviews.

## Conclusion

Our study demonstrates that from a health care perspective the orthoptist-led clinic resulted in a mean reduction in costs per appointment compared to the consultant-led clinic. The difference in cost was driven by the labour costs involved in each clinic.

Health care carries a significant cost across all societies and this study highlights a gain in technical efficiency (i.e., clinical throughput). We believe this study can help future health policy decision making for the allocation of ophthalmic screening resources.

## Additional File

The additional file for this article can be found as follows:

10.22599/bioj.288.s1 Supplementary Materials A. NF1 Orthoptist Led Eye Screening Clinic Guidelines.

## References

[1] Atik, A, Barton, K, Azuara-Blanco, A and Kerr, NM. 2020. Health economic evaluation in ophthalmology. British Journal of Ophthalmology, bjophthalmol-2020-316880. DOI: 10.1136/bjophthalmol-2020-316880 32829299

[2] Ballantyne, M, Liscumb, L, Brandon, E, Jaffar, J, Macdonald, A and Beaune, L. 2019. Mothers’ perceived barriers to and recommendations for health care appointment keeping for children who have cerebral palsy. Global Qualitative Nursing Research, 6: 2333393619868979. DOI: 10.1177/2333393619868979 PMC6696835 31453266

[3] Blazo, MA, Lewis, RA, Chintagumpala, MM, Frazier, M, Mccluggage, C and Plon, SE. 2004. Outcomes of systematic screening for optic pathway tumors in children with neurofibromatosis type 1. American Journal of Medical Genetics Part A, 127A: 224–229. DOI: 10.1002/ajmg.a.20650 15150770

[4] Bouraoui, A, Roberts, L, Landells, A, Law, E, Geh, V, Shrivastava, A and Borg, F. 2020. P16 Orthoptic led eye screening co-located within the paediatric rheumatology clinic for safer management of children with JIA. Rheumatology, 59: Issue Supplement_2. DOI: 10.1093/rheumatology/keaa111.015

[5] Caen, S, Cassiman, C, Legius, E and Casteels, I. 2015. Comparative study of the ophthalmological examinations in neurofibromatosis type 1. Proposal for a new screening algorithm. European Journal of Paediatric Neurology, 19: 415–422. DOI: 10.1016/j.ejpn.2015.03.002 25797697

[6] Campbell, MK and Torgerson, DJ. 1999. Bootstrapping: Estimating confidence intervals for cost-effectiveness ratios. QJM: An International Journal of Medicine, 92: 177–182. DOI: 10.1093/qjmed/92.3.177 10326078

[7] Campen, CJ and Gutmann, DH. 2018. Optic pathway gliomas in neurofibromatosis type 1. Journal of Child Neurology, 33: 73–81. DOI: 10.1177/0883073817739509 29246098 PMC5739070

[8] Cimino, PJ and Gutmann, DH. 2018. Neurofibromatosis type 1. Handbook of Clinical Neurology, 148: 799–811. DOI: 10.1016/B978-0-444-64076-5.00051-X 29478615

[9] Evans, DG, Howard, E, Giblin, C, Clancy, T, Spencer, H, Huson, SM and Lalloo, F. 2010. Birth incidence and prevalence of tumor-prone syndromes: Estimates from a UK family genetic register service. American Journal of Medical Genetics Part A, 152: 327–332. DOI: 10.1002/ajmg.a.33139 20082463

[10] Fair Work Commission Australia. 2016. Nurses and Midvies (Victorian Public Sector) (Single interest Employers) Enterprise Agreement 2016–2020 [Online]. Available at https://www.rch.org.au/uploadedFiles/Main/Content/hr/awards-agreements-entitlements/Nurses%20and%20Midwives%20(Victorian%20Public%20Health%20Sector)(Single%20Interest%20Employers)%20Enterprise%20Agreement%202016%20-%202020.pdf [Accessed April 15 2021].

[11] Fair Work Commission Australia. 2017. Allied Health Professionals (Victorian Public Health Sector) Single Interest Enterprise Agreement 2016–2020 [Online]. Available at https://www.rch.org.au/uploadedFiles/Main/Content/hr/awards-agreements-entitlements/Health%20Professionals%20Interest%20Enterprise%20Agreement%202016%20-%202020.pdf [Accessed April 14 2021].

[12] Fair Work Commission Australia. 2018. AMA Victoria- Victorian Public Health Sector- Medical Specialists Enterprise Agreement 2018–2021 [Online]. Available at https://www.rch.org.au/uploadedFiles/Main/Pages/hr/employee-relations/Medical%20Spedialists%20Enterprise%20Agreement%202018-2021.pdf [Accessed April 14 2020].

[13] Fisher, JM, Avery, AR, Allen, CJ, Ardern-Holmes, LS, Bilaniuk, TL, Ferner, ER, Gutmann, HD, Listernick, JR, Martin, TS, Ullrich, TN and Liu, TG. 2013. Functional outcome measures for NF1-associated optic pathway glioma clinical trials. Neurology, 81: S15–S24. DOI: 10.1212/01.wnl.0000435745.95155.b8 24249802 PMC3908337

[14] Fisher, MJ, Loguidice, M, Gutmann, DH, Listernick, R, Ferner, RE, Ullrich, NJ, Packer, RJ, Tabori, U, Hoffman, RO, Ardern-Holmes, SL, Hummel, TR, Hargrave, DR, Bouffet, E, Charrow, J, Bilaniuk, LT, Balcer, LJ and Liu, GT. 2012. Visual outcomes in children with neurofibromatosis type 1–associated optic pathway glioma following chemotherapy: A multicenter retrospective analysis. Neuro-Oncology, 14: 790–797. DOI: 10.1093/neuonc/nos076 22474213 PMC3367846

[15] Fried, I, Tabori, U, Tihan, T, Reginald, A and Bouffet, E. 2013. Optic pathway gliomas: A review. CNS oncology, 2: 143–159. DOI: 10.2217/cns.12.47 25057976 PMC6169473

[16] Gammie, T, Vogler, S and Babar, Z-U-D. 2016. Economic evaluation of hospital and community pharmacy services. Annals of Pharmacotherapy, 51: 54–65. DOI: 10.1177/1060028016667741 27586430

[17] Guillamo, J-S, Créange, A, Kalifa, C, Grill, J, Rodriguez, D, Doz, F, Barbarot, S, Zerah, M, Sanson, M, Bastuji-Garin, S and Wolkenstein, P. 2003. Prognostic factors of CNS tumours in neurofibromatosis 1 (NF1): A retrospective study of 104 patients. Brain: A Journal of Neurology, 126: 152. DOI: 10.1093/brain/awg016 12477702

[18] Karthikeyan, A, Harthan, S, Mallanaphy, C and Kenawy, N. 2021. Real-world outcomes of allied health professional-led clinic model for assessing and monitoring ocular melanocytic lesions. Eye (London, England), 35: 464–469. DOI: 10.1038/s41433-020-0873-5 32317788 PMC8026979

[19] King, A, Listernick, R, Charrow, J, Piersall, L and Gutmann, DH. 2003. Optic pathway gliomas in neurofibromatosis type 1: The effect of presenting symptoms on outcome. American Journal of Medical Genetics Part A, 122: 95–99. DOI: 10.1002/ajmg.a.20211 12955759

[20] Kirkwood, BJ, Coster, DJ and Essex, RW. 2006. Ophthalmic nurse practitioner led diabetic retinopathy screening. Results of a 3-month trial. Eye (London, England), 20: 173–7. DOI: 10.1038/sj.eye.6701834 16254596

[21] Kleihues, NP, Louis, WD, Scheithauer, BB, Rorke, CL, Reifenberger, KG, Burger, KP and Cavenee, KW. 2002. The WHO classification of tumors of the nervous system. JNEN: Journal of Neuropathology & Experimental Neurology, 61: 215–225. DOI: 10.1093/jnen/61.3.215 11895036

[22] Listernick, R, Charrow, J, Greenwald, M and Mets, M. 1994. Natural history of optic pathway tumors in children with neurofibromatosis type 1: A longitudinal study. The Journal of Pediatrics, 125: 63–66. DOI: 10.1016/S0022-3476(94)70122-9 8021787

[23] Listernick, R, Louis, D, Packer, R and Gutmann, D. 1997. Optic pathway gliomas in children with neurofibromatosis 1: Consensus statement from the NF1 optic pathway glioma task force. American Journal of Ophthalmology, 124: 274–274. DOI: 10.1002/ana.410410204 9029062

[24] Lopetegui, M, Yen, P-Y, Lai, A, Jeffries, J, Embi, P and Payne, P. 2014. Time motion studies in healthcare: What are we talking about? Journal of Biomedical Informatics, 49: 292–299. DOI: 10.1016/j.jbi.2014.02.017 24607863 PMC4058370

[25] Philip, K. 2015. Allied health: Untapped potential in the Australian health system. Australian Health Review, 39: 244–7. DOI: 10.1071/AH14194 26629583

[26] Pokorny, MA, Thorne, PR, Whitfield, BCS, Lee, AC and Wilson, WJ. 2021. Can an advanced audiology-led service reduce waiting times for paediatric ear nose and throat outpatient services? Journal of Paediatrics and Child Health, 57: 268–272. DOI: 10.1111/jpc.15218 33043535

[27] Purchasing Power Parities (Ppp) (Indicator) [Online]. Available at https://data.oecd.org/conversion/purchasing-power-parities-ppp.htm [Accessed 23 August 2020].

[28] Sandinha, T, Hebbar, G, Kenawy, N, Hope-Stone, L and Damato, B. 2012. A nurse-led ocular oncology clinic in Liverpool: Results of a 6-month trial. Eye (London, England), 26: 937–943. DOI: 10.1038/eye.2012.62 22522723 PMC3396164

[29] Stute, M, Moretto, N, Waters, R, Raymer, M, Sam, S, Bhagwat, M, Banks, M, Comans, T and Buttrum, P. 2021. Allied health primary contact services: Results of a 2-year follow-up study of clinical effectiveness, safety, wait times and impact on medical specialist out-patient waitlists. Australian Health Review. DOI: 10.1071/AH19225 33271059

[30] Suharwardy, J and Elston, J. 1997. The clinical presentation of children with tumours affecting the anterior visual pathways. Eye (London, England), 11(pt 6): 838–844. DOI: 10.1038/eye.1997.215 9537141

[31] Thiagalingam, S, Flaherty, M, Billson, F and North, K. 2004. Neurofibromatosis type 1 and optic pathway gliomas: Follow-up of 54 patients. Ophthalmology, 111: 568–577. DOI: 10.1016/j.ophtha.2003.06.008 15019338

[32] Tram, JS, Golding, BM, Lim, C, Kuschel, CA and Elder, JE. 2021. Changed ophthalmic workload following introduction of digital retinal photography for retinopathy of prematurity screening. Clinical & Experimental Ophthalmology, 49: 368–372. DOI: 10.1111/ceo.13926 33788997

[33] Watts, CG, Cust, AE, Menzies, SW, Coates, E, Mann, GJ and Morton, RL. 2015. Specialized surveillance for individuals at high risk for melanoma: A cost analysis of a high-risk clinic. JAMA Dermatology, 151: 178–86. DOI: 10.1001/jamadermatol.2014.1952 25389712

[34] Xu, X, Grossetta Nardini, HK and Ruger, JP. 2014. Micro-costing studies in the health and medical literature: Protocol for a systematic review. Systematic Reviews, 3: 47. DOI: 10.1186/2046-4053-3-47 24887208 PMC4036677

