# Supplementary material for: Cost Analysis of Orthoptist-Led Neurofibromatosis Type 1 Screening Clinics
Source: Br Ir Orthopt J. 2023 Apr 10;19(1):26–34. doi: 10.22599/bioj.288 (PMC10103737; doi:10.22599/bioj.288)
Supplement: Supplementary Materials A. — NF1 Orthoptist Led Eye Screening Clinic Guidelines. [file bioj-19-1-288-s1.pdf]

NF1 Orthoptist Led Eye Screening Clinic Guidelines

The Royal Children's Hospital Melbourne

PATIENT GROUP (Eligibility criteria)
Confirmed NF1 Diagnosis
No known Optic Nerve Glioma

REVIEWS
Will be requested by NF clinic team member
6monthly r/v until age 6
12 monthly r/v from age 6

PROTOCOL
Orthoptist-led clinic. Registrar is rostered on if needed.
VA
Pupils
Fundus – un-dilated photo– Big Pupil/low flash setting as ON most important. If photo not possible, dilated fundus examination to be performed by doctor
OCT – RNFL – un-dilated

If unable to obtain VA and/or Pupils and/or Fundus – orthoptist to order consultant clinic review NF Screening clinic when cooperation improves. NF coordinator to flag this patient group to ensure follow up is in appropriate clinic.

NORMAL FINDINGS
Continue r/v in NF screening clinic as per above at request of NF clinic
Discharge as per NF clinic. (Approximately age 18)

ABNORMAL FINDINGS
VA – difference in VA between RE and LE or reduced VA
PUPILS – any RAPD
PHOTO – any optic disc changes (Initially to be confirmed by Fellow)
OCT – RNFL changes (Initially to be confirmed by Fellow)
Appointment given in Consultant-led Eye General/Genetic clinic.
